# Dietary saturated fatty acids and prostate cancer: insights into NF-κB pathway and lipid metabolism mechanisms

**DOI:** 10.1007/s12672-025-03005-0

**Published:** 2025-06-20

**Authors:** Jia Wei He, Zi Xuan Huang, Yan Feng Su, Yang Qi Mo

**Affiliations:** Dongguan Songshan Lake Central Hospital, Dongguan, 523320 China

**Keywords:** Prostate cancer, Saturated fatty acids, NF-κB pathway, Dietary factors, Cancer progression.

## Abstract

Prostate cancer (PCa) is one of the most prevalent malignancies in men, with dietary factors, particularly saturated fatty acids (SFAs), emerging as significant contributors to its pathogenesis. This review synthesizes current literature linking dietary fat intake, especially from dairy products, to increased prostate cancer risk, highlighting specific SFAs such as myristic and palmitic acids. The nuclear factor kappa-light-chain-enhancer of activated B cells (NF-κB) signaling pathway is identified as a critical mediator in this relationship, promoting cell survival and proliferation in the tumor microenvironment. Additionally, the role of the lipogenic enzyme ELOVL7 is explored, emphasizing its involvement in fatty acid metabolism and cancer progression. While epidemiological and mechanistic studies provide compelling evidence for the link between dietary SFAs and prostate cancer through NF-κB activation, significant knowledge gaps remain, necessitating further research to elucidate the underlying mechanisms and potential therapeutic strategies targeting lipid metabolism.

## Introduction

Prostate cancer (PCa) represents one of the most prevalent forms of cancer among men globally, with its incidence rates increasing significantly in recent years. Several factors contribute to its pathogenesis, including genetic predispositions, environmental influences, and dietary habits. Among these, dietary fat intake, particularly saturated fatty acids (SFAs), has emerged as a critical factor associated with prostate cancer development and progression. Epidemiological studies have consistently shown a correlation between high consumption of dairy products and increased risk of prostate cancer, suggesting that SFAs may play a role in this association [1].

The nuclear factor kappa-light-chain-enhancer of activated B cells (NF-κB) signaling pathway has been implicated in the progression of various cancers, including prostate cancer. NF-κB is a transcription factor that regulates the expression of genes involved in inflammation, cell proliferation, and survival. In the context of prostate cancer, the activation of the NF-κB pathway has been associated with tumor progression, metastasis, and resistance to apoptosis [2]. This pathway is often activated in response to inflammatory cytokines, which can be produced in the tumor microenvironment, further supporting the link between diet, inflammation, and cancer progression.

Recent studies have suggested that SFAs can activate the NF-κB signaling pathway, thereby contributing to prostate cancer progression. For instance, the expression of specific enzymes involved in fatty acid metabolism, such as fatty acid synthase (FASN), has been shown to correlate with NF-κB activity and cancer aggressiveness [3]. High-fat diets have been demonstrated to promote the growth of tumors in models of prostate cancer, indicating that dietary fats may influence cancer biology through mechanisms involving NF-κB activation [4].

The significance of this study lies in its potential to unravel the mechanisms by which saturated fatty acids contribute to prostate cancer progression through the activation of the NF-κB pathway. By elucidating these pathways, we can identify potential therapeutic targets and preventive strategies aimed at reducing the risk of prostate cancer associated with dietary fat intake. Understanding the interplay between diet, inflammation, and cancer biology is crucial for developing effective interventions that can mitigate the rising incidence of prostate cancer globally.

## Diet, NF-κB and prostate cancer insights

### Dietary fats and prostate cancer

The association between dietary fats and the risk of prostate cancer has been a significant research focus. Epidemiological studies have shown a strong link between high intake of saturated fatty acids (SFAs) and increased risk of prostate cancer. A noteworthy study conducted in Japan highlighted a dose-dependent relationship between dairy product consumption, which is high in saturated fats, and the risk of prostate cancer, reporting relative risks of 1.63 for total dairy products and 1.62 for myristic acid specifically. This suggests that certain SFAs may play a crucial role in prostate carcinogenesis.

Further research has indicated that lipid metabolism is integral to prostate cancer progression. Tamura et al. (2009) identified the ELOVL7 gene, which encodes a long-chain fatty acid elongase, as being overexpressed in prostate cancer cells. This gene is regulated by the androgen signaling pathway and is involved in synthesizing saturated very-long-chain fatty acids (SVLFAs) which promote cancer cell growth [5]. The findings suggest that dietary fat, particularly saturated fat, not only influences cancer risk but also may impact tumor growth dynamics.

### NF-κB signaling pathway

The nuclear factor kappa-light-chain-enhancer of activated B cells (NF-κB) signaling pathway is critically involved in the regulation of inflammatory responses and cancer progression. In prostate cancer, NF-κB activation is associated with increased cell survival and proliferation. For instance, Shankar et al. (2017) reported that betulinic acid, a potential therapeutic compound, induces apoptosis in prostate cancer cells by inhibiting NF-κB signaling, thereby preventing its translocation to the nucleus [6].

The role of NF-κB in cancer is further supported by the findings of Paschke et al. (2016), which demonstrated that ZFP91, an oncogenic protein, activates the noncanonical NF-κB pathway, suggesting a complex interplay between various signaling mechanisms in prostate cancer biology [7]. The activation of NF-κB has been implicated in the metastatic potential of prostate cancer, highlighting its importance as a target for therapeutic intervention [8].

### Interaction between dietary fats and NF-κB signaling

The interplay between dietary fats and the NF-κB signaling pathway is gaining attention in understanding prostate cancer progression. Excessive accumulation of saturated fatty acids has been linked with the activation of NF-κB, which in turn promotes tumorigenesis. Wang et al. (2022) found that higher levels of apolipoproteins and triglycerides in the blood of prostate cancer patients correlate with increased expression of enzymes involved in lipid metabolism, such as FASN and ACC1, which are known to activate NF-κB signaling [9].

Furthermore, dietary fat intake appears to influence the inflammatory microenvironment of tumors, with high-fat diets supporting an inflammatory state conducive to cancer progression [10]. This underscores the necessity of further research to elucidate the molecular mechanisms by which dietary fat influences NF-κB activity and contributes to prostate cancer pathogenesis.

In summary, the interaction between dietary fats and the NF-κB signaling pathway represents a critical area of study in understanding the mechanisms underlying prostate cancer progression and highlights potential avenues for therapeutic intervention.

## Unveiling the roles of dietary components and key molecules

### Association between dairy products and prostate cancer

Further exploration into the dietary fat component revealed significant insights into the relationship between saturated fatty acids and prostate carcinogenesis. Chavarro et al. (2013) examined the blood levels of specific saturated and monounsaturated fatty acids in prediagnostic samples from prostate cancer cases [11]. Their results indicated that higher blood levels of the fatty acid 16:1n-7 were associated with an increased incidence of high-grade prostate tumors (Gleason score ≥ 7), suggesting that de novo lipogenesis, reflected by blood fatty acid levels, may play a role in the progression of prostate cancer. This underscores the importance of dietary fat intake in prostate cancer risk and highlights the potential for further research into dietary modifications as preventive strategies.

In addition to the findings mentioned above, a study focusing on the impact of a high-fat diet (HFD) on ELOVL7-expressing prostate cancer cells was conducted. When compared to cells on a normal diet (ND), those exposed to an HFD exhibited a differential growth pattern, suggesting that HFD may promote the growth of ELOVL7-expressing prostate cancer cells. To further explore this relationship, a colony formation assay was performed on 22Rv1 and LNCaP cells transfected with shRNA-expressing vectors targeting ELOVL7. Under HFD conditions, a trend towards increased cell growth was observed, with a significance level of *P* = 0.191. Although this P-value does not reach the conventional threshold for statistical significance (*P* < 0.05), it indicates a potential tendency. It is also worth noting that this result may be influenced by the ectopic expression of ELOVL7, which can vary among different cell clones. Collectively, while the statistical significance of the effect of HFD on the growth of these prostate cancer cells is not highly conclusive in this study, the observed trend, combined with the known role of ELOVL7 in lipid metabolism and prostate cancer progression, provides valuable insights. These findings further emphasize the complex role of dietary fat in modulating the behavior of prostate cancer cells and suggest that further research is warranted to elucidate this relationship more precisely.

### Molecular mechanisms of biseugenol B

Biseugenol B has emerged as a promising candidate for prostate cancer treatment due to its apoptotic effects on cancer cells. Abbaspour Babaei et al. (2017) investigated the molecular mechanisms underlying the cytotoxic effects of biseugenol B on the human prostate cancer cell line PC3. The study revealed that treatment with biseugenol B led to significant changes in cell metabolism, including nuclear condensation, altered mitochondrial membrane potential (MMP), and cytochrome c release. The treatment also resulted in the downregulation of the anti-apoptotic protein Bcl-2 and upregulation of the pro-apoptotic Bax protein, which facilitated apoptotic signaling. Caspase activation was noted as well, indicating the involvement of both intrinsic and extrinsic apoptotic pathways. Importantly, the study found that biseugenol B inhibited the translocation of NF-κB from the cytosol to the nucleus in PC3 cells, suggesting a dual mechanism of apoptosis induction and NF-κB signaling inhibition. These findings position biseugenol B as a potentially effective therapeutic agent for targeting prostate cancer (Abbaspour Babaei et al., 2017).

Moreover, Eswar Shankar et al. (2017) demonstrated that defects in p53 and NF-κB signaling pathways are frequently observed in the initiation and development of various human malignancies, including prostate cancer. Their research showed higher expression of NF-κB/p65/RelA, NF-κB/p50/RelB, and cRel, as well as downregulation of the p53 network in primary prostate cancer specimens and in metastatic tumors. This indicates that targeting the NF-κB pathway could provide a therapeutic avenue for prostate cancer treatment, potentially complementing the effects of compounds like biseugenol B (Eswar Shankar et al., 2017).

### Role of ELOVL7 in lipid metabolism

ELOVL7 has been identified as a crucial player in the lipid metabolism associated with prostate cancer growth. Tamura et al. (2009) highlighted the overexpression of ELOVL7 in prostate cancer cells, proposing its role as a long-chain fatty acid elongase. Their research demonstrated that the knockdown of ELOVL7 markedly reduced prostate cancer cell growth, while a high-fat diet significantly promoted tumor growth in ELOVL7-expressing prostate cancer models. The study also provided evidence that ELOVL7 preferentially elongates saturated very-long-chain fatty acids (SVLFAs), which are implicated in lipid metabolism and androgen synthesis within prostate cancer cells. This suggests that ELOVL7 not only contributes to cancer cell proliferation but may also mediate the effects of dietary fat intake on prostate carcinogenesis (Tamura et al., 2009).

In a follow-up analysis, the impact of ELOVL7 knockdown on the fatty acid composition of C4-2B cells was assessed through GC/MS. The results showed a specific reduction of saturated very-long-chain fatty acids (SVLFA C20:0, C22:0, C24:0), indicating that ELOVL7 plays a significant role in regulating lipid profiles in prostate cancer cells. This is consistent with the findings in LNCaP cells where ELOVL7 knockdown led to similar fatty acid composition changes.

The implications of dietary fat and ELOVL7 expression extend beyond mere cancer cell growth; they also encompass metabolic pathways that influence androgen synthesis. In a follow-up analysis, ELOVL7’s role in altering lipid profiles and its connection to de novo androgen synthesis were further elucidated, indicating that ELOVL7 could be a pivotal target for therapeutic strategies aimed at mitigating prostate cancer progression via dietary interventions (Tamura et al., 2009).

### Prostate cancer diet molecule NF-κB research gaps

The studies presented in this section offer valuable insights into the roles of dietary components and key molecules in the context of prostate cancer. Regarding the association between dairy products and prostate cancer, research indicates that dietary fat intake, particularly from dairy sources, may be linked to an increased risk of prostate cancer, with blood fatty acid levels potentially playing a role in tumor progression. The therapeutic potential of Biseugenol B in prostate cancer treatment is promising, as it has been shown to induce apoptosis in cancer cells and inhibit the NF-κB signaling pathway. Additionally, ELOVL7 has been identified as a crucial factor in lipid metabolism related to prostate cancer growth, influencing both fatty acid composition and androgen synthesis. However, while these findings are significant, they have not yet explored in depth how these factors specifically affect the risk of prostate cancer through the NF-κB pathway. The NF-κB pathway is known to be involved in various aspects of cancer development; however, the exact mechanisms by which dairy products, Biseugenol B, and ELOVL7 interact with this pathway remain unclear.

## Major findings

### Epidemiological evidence

**Fig. 1:**
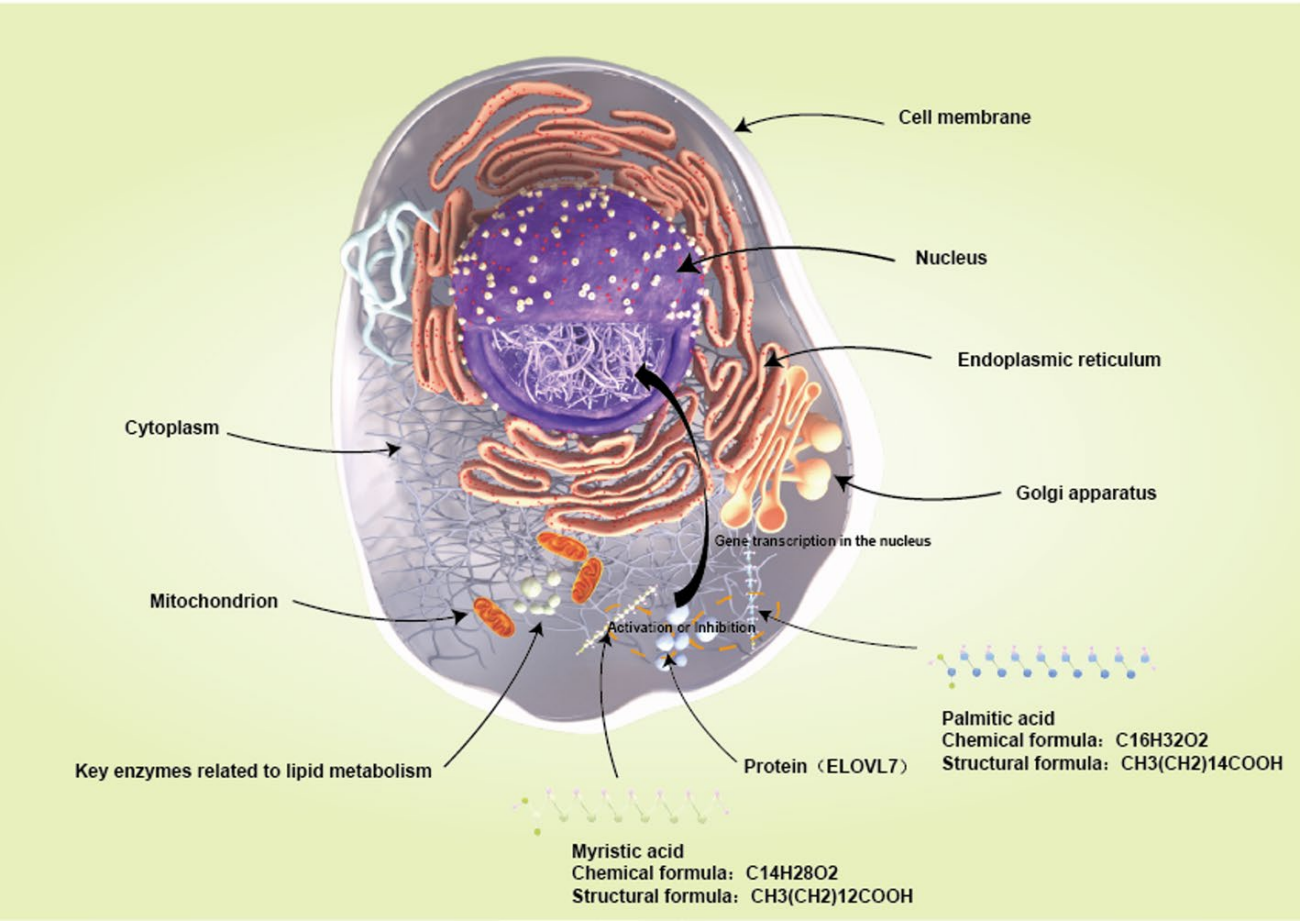
Metabolic association diagram of saturated fatty acids and ELOVL7 in prostate cancer cells

Numerous epidemiological studies have established a positive association between dietary fat intake, particularly saturated fatty acids, and the incidence of prostate cancer. For example, Kurahashi et al. (2008) conducted a study involving 43,435 Japanese men aged 45–74 years. They found a dose - dependent increase in prostate cancer risk with dairy product consumption. The relative risks for the highest versus the lowest quartiles of total dairy products, milk, and yogurt were 1.63 (1.14–2.32), 1.53 (1.07–2.19), and 1.52 (1.10–2.12), respectively. Specific saturated fatty acids, like myristic and palmitic acids, had relative risks of 1.62 (1.15–2.29) and 1.53 (1.07–2.20), respectively, for increasing prostate cancer risk. However, controlling for confounding factors attenuated the relationships for calcium and saturated fatty acids, indicating a complex dietary influence on prostate cancer incidence. Tamura et al. (2009) identified the lipogenic gene ELOVL7 as overexpressed in prostate cancer cells. This suggests that lipid metabolism, specifically the metabolism of saturated very - long - chain fatty acids (SVLFAs) by ELOVL7, plays a role in prostate carcinogenesis, emphasizing the link between dietary fat intake and cancer development.(Fig. 1).

Furthermore, research shows that excessive accumulation of saturated fatty acids and cholesterol may contribute to prostate cancer [12–20]. Wang et al. (2022) found that lipoproteins and triglycerides were elevated in prostate cancer patients’ blood, and key lipid - metabolism - related enzymes (ACC1, FASN, HMGCR) were highly expressed in cancer tissues, positively correlating with the Gleason score. Paschke et al. (2016) identified ZFP91, a zinc finger protein upregulated in prostate cancer cell lines, which is involved in activating the noncanonical NF - κB signaling pathway and HIF − 1α signaling, highlighting its relevance in prostate cancer biology.

### Mechanistic insights

**Fig. 2:**
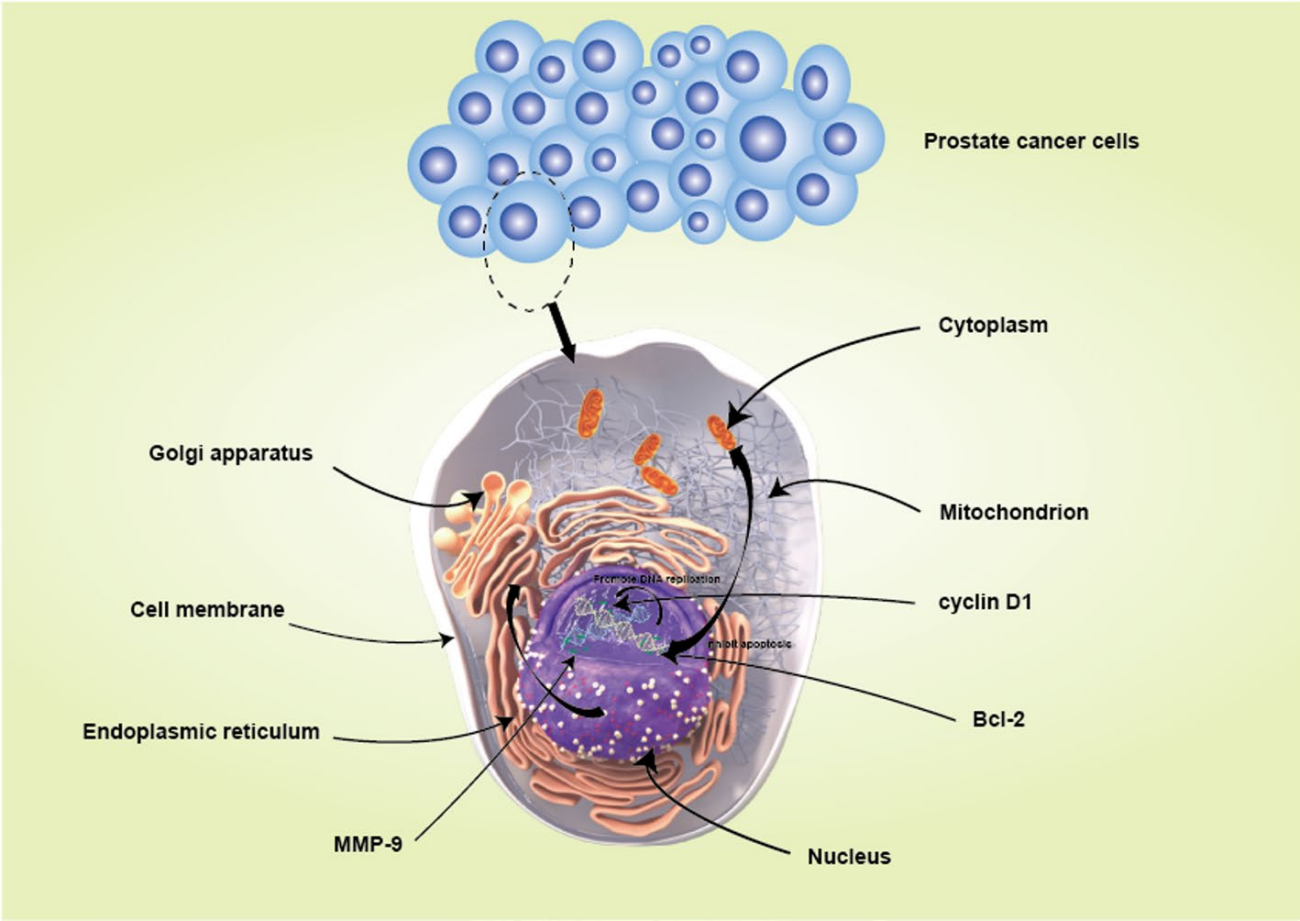
Schematic diagram of NF - κ B pathway and apoptosis related proteins in prostate cancer cells

The molecular mechanisms linking dietary fat intake to prostate cancer progression have been explored in various studies, with a particular focus on the NF-κB signaling pathway and apoptosis. The NF-κB pathway is known to promote tumor cell survival through anti-apoptotic signaling, and its activation is frequently observed in prostate cancer. For instance, Eswar Shankar et al. (2017) noted that defects in the NF-κB signaling pathway are commonly found in prostate cancer, leading to increased cell survival and potential metastasis(Fig. 2). Their research also indicated that betulinic acid could induce apoptosis by stabilizing p53 and downregulating NF-κB activity, underscoring the importance of this pathway in cancer therapy.

Moreover, studies have demonstrated that specific compounds can trigger apoptosis in prostate cancer cells through the NF-κB pathway [21–30]. For example, biseugenol B was found to induce apoptosis in PC3 prostate cancer cells by inhibiting NF-κB translocation and altering the Bax/Bcl-2 ratio, leading to cytochrome c release and subsequent caspase activation (Abbaspour Babaei et al., 2017). This indicates that targeting NF-κB may provide a therapeutic strategy in managing prostate cancer.

In another study, the traditional medicinal plant Phyllanthus showed the ability to interfere with multiple signaling pathways involved in tumorigenesis, including the NF-κB pathway, suggesting its potential as a therapeutic candidate for prostate cancer (Tang et al., 2013). Additionally, recent research by Nguyen et al. (2014) discussed the role of inflammation and NF-κB activation in prostate cancer progression, highlighting the need for further exploration of inflammatory pathways to develop novel therapeutic strategie. Overall, these findings highlight the critical role of the NF-κB pathway in the context of prostate cancer progression and suggest that dietary fats may influence this pathway, thereby affecting cancer outcomes(Table 1).

**Table 1:** The role of NF - κ B pathway and lipid metabolism in prostate cancer

References	object	method	NF - κ B pathway and prostate cancer	lipid metabolism and prostate cancer	Key findings	Clinical significance
[2] Nguyen DP et al.	Prostate cancer cells	In vitro cell experiments, animal model experiments, and clinical sample analysis	In prostate cancer, the NF - κ B pathway is abnormally activated and plays an important role in multiple critical stages of disease progression	NA	NF - κ B activation is associated with prostate cancer progression indicators such as Gleason score, biochemical recurrence, and disease-specific survival	Aids in predicting prostate cancer prognosis and developing NF - κ B - targeted therapies for better outcomes
[3] Xiaoying Wang et al.	Prostate cancer cell lines of prostate cancer patients and transgenic mice	Prostate cancer cases and mouse model experiments	NA	The peripheral blood levels of lipoprotein, apolipoprotein, triglycerides, and free fatty acids in prostate cancer patients are significantly higher than those in non cancer patients	Fatty acids and cholesterol synergistically promote the progression of prostate cancer	Suggests regulating lipid metabolism as a prostate cancer preventive/therapeutic strategy
[1] Norie Kurahashi et al.	Japanese male	questionnaire	NA	The intake of saturated fatty acids (such as myristic acid and palmitic acid) in dairy products is dose-dependent and increases the risk of prostate cancer, indicating a correlation between lipid metabolism and prostate cancer	The intake of dairy products increases the risk of prostate cancer in a dose-dependent manner	Provides dietary advice: limit dairy product intake to reduce prostate cancer risk
[8] Maryam Abbaspour Babaei et al.	Human prostate cancer cell line	Cell experiment	Dieugenol B can inhibit NF - κ B translocation induced by tumor necrosis factor - α (TNF - α) in PC3 cells, thereby promoting cancer cell apoptosis	NA	Dieugenol B has significant cytotoxicity towards PC3 cells and low cytotoxicity towards normal RWPE-1 cells, and can induce apoptosis in PC3 cells	Indicates Dieugenol B’s potential as a prostate cancer drug with selective cytotoxicity
[9] Yin-Quan Tang et al.	Human prostate cancer cells	Cell experiment	The abnormal activation of NF - κ B promotes the survival and metastasis of cancer cells, while the extract of Phyllanthus edulis can exert anti-cancer effects by inhibiting key proteins in the NF - κ B pathway	NA	Yexiazhu extract can significantly downregulate the NF - κ B pathway in PC-3 cells to inhibit the progression of prostate cancer	Shows Yexiazhu extract’s potential in developing natural anti - prostate cancer drugs
[6] Lukasz Paschke et al.	Prostate cancer tissue and cell lines	Gene and protein experiments	ZFP91 can stabilize and activate NIK through ubiquitination, thereby affecting the occurrence and development of prostate cancer	NA	ZFP91 protein may accumulate or remain stable in prostate cancer cells	Implies targeting ZFP91 for prostate cancer treatment
[11] Jorge E. Chavarro et al.	Prostate cancer cases	Case control	NA	The generation of de novo fat through palmitoleic acid may be an important metabolic pathway for the development of prostate cancer	The level of palmitoleic acid (16:1n -7) in blood is positively correlated with the incidence rate of prostate cancer	Highlights monitoring palmitoleic acid for prostate cancer prevention
[5] Eswar Shankar et al.	Human prostate cancer cell line	Gene and protein experiments	The p65 subunit of the NF - κ B pathway is associated with the progression, recurrence, and chemotherapy resistance of prostate cancer	NA	Betulinic acid (BA) inhibits the proliferation of prostate cancer cells by downregulating the NF - κ B pathway by reducing the phosphorylation of I κ B kinase (IKK) α and I - κ B - α (I κ B α)	Shows Betulinic acid’s potential in treating prostate cancer via the NF - κ B pathway
[4] Kenji Tamura et al.	Prostate cancer cells and tissues	Gene experiment	NA	Lipid metabolism related genes can affect the metabolism of saturated long-chain fatty acids, cholesterol esters, and androgen synthesis to participate in the progression of prostate cancer	A high-fat diet promotes the growth of prostate cancer cells related to lipid metabolism gene expression in vivo	Emphasizes considering lipid metabolism genes and dietary fat in prostate cancer management

## Analysis of dietary fats and prostate cancer progression

The association between dietary fats, particularly saturated fatty acids, and the progression of prostate cancer has been a subject of extensive research, yielding both consistent and inconsistent findings across various studies. This section critically examines these consistencies and discrepancies, focusing on the mechanisms through which dietary fats may influence prostate cancer progression, especially through the activation of the NF-κB pathway [31–40].

Several epidemiological studies have reported a positive correlation between dairy product consumption and prostate cancer risk, attributing this association to either calcium or saturated fatty acids present in these products. Kurahashi et al. (2008) conducted a prospective study involving 43,435 Japanese men and found that higher intakes of dairy products were linked to a dose-dependent increase in prostate cancer risk. Specific saturated fatty acids, such as myristic and palmitic acids, were observed to significantly elevate the risk, although this association weakened after adjusting for confounding factors [41–50]. This finding suggests that while there is a notable correlation, the role of saturated fatty acids in prostate cancer progression might be complex and influenced by various dietary and lifestyle factors.

Conversely, Tamura et al. (2009) highlighted the importance of lipid metabolism in prostate carcinogenesis, identifying the ELOVL7 gene as being overexpressed in prostate cancer cells. The study indicated that high-fat diets could promote tumor growth in ELOVL7-expressing prostate cancer, suggesting a more direct mechanism linking dietary fats to cancer progression [51–60]. This aligns with findings from Shankar et al. (2017), who noted that alterations in the NF-κB pathway are common in prostate cancer and that dietary fats could exacerbate these changes, leading to increased tumor survival and proliferation.

However, discrepancies arise when considering the effects of different types of fatty acids. For instance, Wang et al. (2022) reported that elevated levels of saturated fatty acids and cholesterol were linked to prostate cancer, yet the precise mechanisms through which these lipids affect cancer progression remain inadequately understood [61–70]. Notably, while some studies suggest that increased saturated fat intake correlates with higher cancer risk, others have not found a direct causative link, indicating that the relationship may be more nuanced than previously thought.

**Fig. 3:**
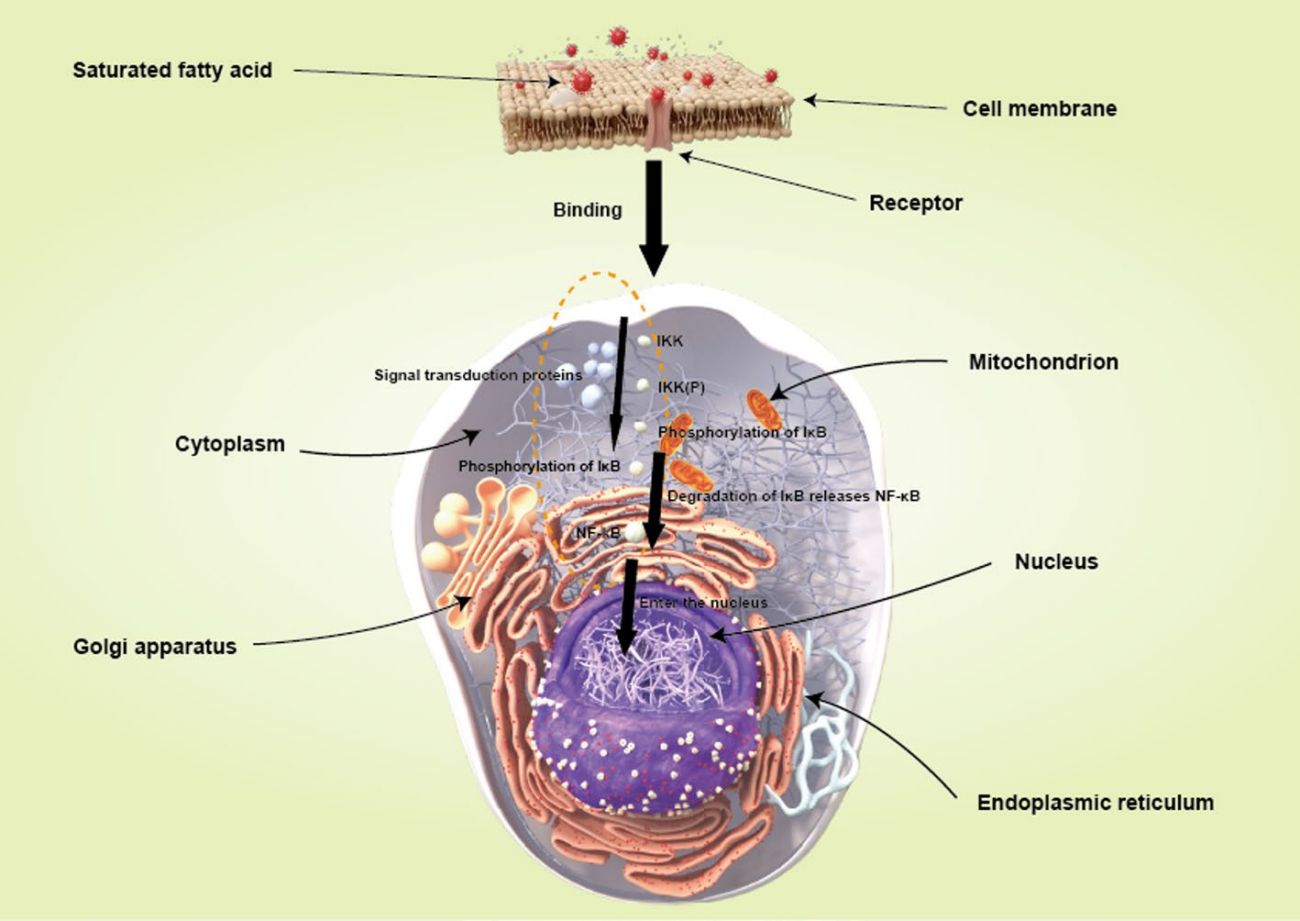
Mechanism diagram of NF - κ B pathway activation in prostate cancer cells by saturated fatty acids

In terms of molecular mechanisms, the NF-κB signaling pathway has been identified as a critical player in prostate cancer progression [71–89]. According to Nguyen et al. (2014), NF-κB activation promotes tumor cell survival through anti-apoptotic signaling, which is often fueled by inflammatory processes associated with high-fat diets(Fig. 3). This pathway’s involvement suggests that dietary fats may not only impact cancer risk but also influence the aggressiveness of the disease once it has developed.

In summary, while there is a consensus among some studies regarding the risk associated with saturated fatty acids and prostate cancer, significant variability exists in the findings, particularly concerning the types of fats and their metabolic pathways. Further research is necessary to clarify the mechanistic underpinnings of these associations, particularly the role of the NF-κB pathway in mediating the effects of dietary fats on prostate cancer progression.

## Research trends and gaps

The role of dietary fats in the progression of prostate cancer has gained considerable attention in recent years. Emerging research indicates a significant association between the intake of saturated fatty acids (SFAs) and the development of prostate cancer, particularly through the activation of the NF-κB signaling pathway. This section will discuss the current trends in this research area and highlight knowledge gaps that need to be addressed.

### Emerging research trends

Recent studies have underscored the importance of dietary fats, particularly SFAs, in the etiology of prostate cancer. For instance, Kurahashi et al. (2008) found a dose-dependent relationship between dairy product consumption, which is high in saturated fats, and prostate cancer risk in a Japanese cohort. Their findings suggest that specific fatty acids, such as myristic and palmitic acid, significantly elevate the risk of developing prostate cancer.

In another study, Tamura et al. (2009) identified ELOVL7, a gene involved in fatty acid elongation, as being overexpressed in prostate cancer cells. This lipogenic gene appears to play a crucial role in the metabolism of saturated very-long-chain fatty acids (SVLFAs), which are implicated in prostate cancer growth and survival. These findings suggest that metabolic pathways related to fatty acid metabolism are critical in understanding prostate cancer progression.

Moreover, Shankar et al. (2017) emphasized the relationship between NF-κB signaling and prostate cancer. The study demonstrated that targeting NF-κB could alter apoptosis pathways in prostate cancer cells. This indicates a potential therapeutic avenue, as manipulating dietary fat intake may influence NF-κB activity and, consequently, cancer progression.

### Identified gaps in knowledge

Despite these advances, several gaps remain in our understanding of the mechanisms linking dietary fats and prostate cancer. First, the specific pathways through which SFAs exert their effects are not fully elucidated. While the involvement of NF-κB has been documented, the interplay between various signaling pathways, including other inflammatory pathways and metabolic processes, remains inadequately explored (Nguyen et al., 2014).

Additionally, the heterogeneity of dietary fat sources and their distinct fatty acid compositions complicates the extrapolation of findings across different populations. For instance, the exact contributions of individual SFAs versus other dietary fats, such as monounsaturated and polyunsaturated fatty acids, require further investigation to ascertain their specific roles in prostate cancer risk (Wang et al., 2022).

Furthermore, most studies have predominantly focused on the correlational aspects of dietary fat intake and prostate cancer incidence, with limited longitudinal data to establish causation. More comprehensive cohort studies are needed to clarify these relationships and determine whether dietary interventions can effectively modify prostate cancer outcomes.

In conclusion, while there is a growing body of evidence linking dietary fats, particularly SFAs, to prostate cancer progression, further research is necessary to unravel the underlying mechanisms and address the existing knowledge gaps. Addressing these gaps may prove vital in developing effective dietary strategies for prostate cancer prevention and treatment.

## Limitations of current research

### Methodological limitations

In epidemiological studies, the large-scale population - based prospective study conducted by Kurahashi et al. in 2008 serves as a prime example. This study heavily relied on self - reported dietary intake via questionnaires, a method that is highly susceptible to recall bias. The inherent fallibility of human memory means that participants may inaccurately recall their food consumption, leading to misclassification of exposure levels. Even when attempts are made to control for confounding factors, it is likely that not all relevant dietary and lifestyle variables are accounted for [90–92]. This oversight can obfuscate the true relationship between dairy intake, SFAs, and prostate cancer risk. To mitigate these issues, integrating biomarkers of fatty acid intake could significantly enhance the accuracy of dietary assessment. For instance, analyzing fatty acid metabolites in blood or urine provides a more objective reflection of an individual’s fatty acid ingestion. Additionally, leveraging digital food - tracking tools, such as certain mobile applications that enable real - time dietary recording, can effectively reduce recall bias and enhance the reliability of research data.

Regarding studies on the NF - κB pathway and its role in prostate cancer progression, exemplified by those of Wang et al. in 2022 and Nguyen et al. in 2014, in vitro cell experiments and animal models are frequently employed. However, these models fall short of fully recapitulating the complexity of human physiology. The interactions among dietary components, cancer cells, and the tumor microenvironment in humans are substantially different from those in laboratory settings, posing a significant challenge when translating research findings into clinical applications. Moreover, the use of specific cell lines like PC3 and LNCaP in these studies further limits the generalizability of the results, as these cell lines may not adequately represent the heterogeneity of prostate cancer observed in patients. To address these limitations, the utilization of patient - derived organoids or xenografts is recommended. Patient - derived organoids preserve the original characteristics of tumor cells and can better reflect tumor heterogeneity, while xenografts can simulate the growth environment of human tumors in animals, providing a more realistic representation of the interactions between tumors and the body [93–96]. This approach is conducive to improving the clinical relevance and translatability of research findings.

In terms of methods for assessing fatty acid composition and its impact on cancer progression, techniques such as gas chromatography and gene expression analysis also have inherent flaws. As noted in the studies by Tamura et al. in 2009 and Shankar et al. in 2017, which identified key lipogenic genes and pathways involved in cancer progression, the specificity and sensitivity of these assays can influence the accuracy of the results. Moreover, relying solely on gene expression data without functional validation restricts the ability to draw definitive conclusions about the biological significance of these findings. In future research, it is essential to complement gene expression analysis with functional validation experiments, such as gene knockout and overexpression studies, to clarify the specific functions of genes in cancer progression and gain a deeper understanding of the relationship between fatty acids and prostate cancer.

### Biological limitations

Biological factors significantly limit the translation of current research findings into clinical practice. Prostate cancer heterogeneity is a crucial aspect that should be recognized as a driving force for precision nutrition research. As emphasized by Paschke et al. (2016), the expression of markers such as ZFP91 can vary markedly among different patients. This heterogeneity challenges the establishment of a universal approach to prostate cancer treatment or prevention based on dietary fat intake. Instead, it highlights the need for personalized strategies that take into account individual differences in cancer pathology and responses to dietary factors. To this end, future studies should stratify participants according to genetic or molecular tumor profiles. By doing so, researchers can better understand how different subgroups of patients respond to dietary interventions, leading to more targeted and effective nutrition - based preventive and therapeutic approaches.

Most of the reviewed studies concentrate on specific saturated fatty acids, like myristic and palmitic acids, while neglecting the broader context of dietary patterns and the complex interplay between various fatty acids and other dietary components. The biological impact of dietary fats is not determined by individual fatty acids alone but is rather influenced by the overall diet. For instance, the presence of antioxidants, fiber, and other bioactive compounds can modulate cancer risk. Therefore, isolating the effects of individual fatty acids may not accurately represent their role within the complex dietary matrix that affects prostate cancer progression [97–99]. To overcome this limitation, it is recommended that researchers adopt systems biology approaches to conduct holistic dietary pattern analyses. These approaches can comprehensively analyze how multiple nutrients interact with tumor - promoting pathways such as NF - κB. By integrating data from various - omics technologies, including genomics, proteomics, and metabolomics, researchers can gain a more in - depth understanding of the complex relationships between diet and cancer biology. This integrative approach will provide a more accurate picture of how different dietary components work together to influence prostate cancer development and progression.

Furthermore, the role of inflammation and its interaction with dietary components remain an area in need of further exploration. Inflammation is a critical factor in the development and progression of prostate cancer, with the NF - κB signaling pathway playing a central role in mediating this process. However, the complex interplay between dietary SFAs, inflammation, and cancer biology is not yet fully elucidated. Future research should focus on disentangling these relationships. This could involve in - depth investigations of how dietary SFAs trigger or modulate inflammatory responses, and how these responses, in turn, interact with NF - κB and other cancer - related pathways. Understanding these complex interactions is essential for developing evidence - based dietary recommendations and targeted therapies for prostate cancer patients.

## Future research directions

The relationship among saturated fatty acids (SFAs), the NF - κB signaling pathway, and prostate cancer (PCa) progression is complex and not fully understood, presenting a fertile area for in - depth exploration. Epidemiological studies, such as the one by Kurahashi et al. (2008), have firmly established a positive correlation between dietary fat intake, especially from dairy products, and an increased risk of prostate cancer. Their research indicates that specific SFAs, like myristic and palmitic acids, play a role in elevating this risk. However, significant gaps exist in our knowledge regarding the underlying molecular mechanisms.

### Gaps in current knowledge

Firstly, while it is established that saturated fatty acids (SFAs) can activate the NF-κB pathway, the precise molecular mechanisms and intermediary molecules involved in this activation process within prostate cancer cells remain unclear. Furthermore, the interaction between SFAs and other signaling pathways that may influence NF-κB activation, particularly those associated with inflammation and lipid metabolism, has not been thoroughly investigated. Secondly, the variability in the effects of different SFAs on NF-κB activation and prostate cancer progression is inadequately characterized. Distinct SFAs may operate through unique mechanisms, and comprehending these differences is essential for developing targeted prevention and treatment strategies. Lastly, most existing studies lack a holistic approach that integrates multiple biological data levels, such as lipidomic, transcriptomic, and proteomic information, to fully elucidate the intricate relationship between SFAs, NF-κB, and prostate cancer.

### Specific testable research questions and advanced research methodologies

Based on the current knowledge gaps in the research on the relationship among saturated fatty acids (SFAs), the nuclear factor kappa-B (NF-κB) signaling pathway, and prostate cancer, a series of specific and testable research questions can be proposed and explored using advanced research methods. In prostate cancer cells, the interaction mechanisms between specific SFAs (such as myristic acid and palmitic acid) and cell membrane receptors or intracellular proteins, as well as the specific processes by which this interaction initiates the activation of the NF-κB pathway, remain unclear. Research is needed to identify the interaction sites and modes between these specific SFAs and related molecules to elucidate the initial steps of NF-κB pathway activation [100–103]. The lipogenic enzyme ELOVL7 is overexpressed in prostate cancer cells; however, the impact of its mediated fatty acid elongation process on the activation of the NF-κB pathway by SFAs requires further investigation. Additionally, it is necessary to explore the changes in NF-κB-regulated gene expression under SFA exposure after inhibiting ELOVL7. Clarifying these issues will contribute to a deeper understanding of the role of lipogenic enzymes in the development and progression of prostate cancer. The release of inflammatory cytokines is triggered by SFA exposure; however, the precise regulatory mechanisms of these inflammatory cytokines on the activation of the NF-κB pathway in prostate cancer cells remain unclear. Identifying specific cytokine-NF-κB regulatory loops is of great significance for the development of therapeutic interventions for prostate cancer.

To address the aforementioned questions, advanced research methods can be employed. Lipidomic analysis technology can be utilized to investigate the alterations in lipid species in prostate cancer cells treated with saturated fatty acids (SFAs). By quantifying the levels of various SFAs and their metabolites, potential lipid mediators implicated in NF-κB activation can be identified. Analyzing the correlation between shifts in the levels of lipid second messengers and the activation status of NF-κB can provide valuable insights into the early events of the SFA-NF-κB signaling cascade. Transcriptomic techniques, such as RNA sequencing, can be leveraged to analyze the global changes in gene expression in prostate cancer cells exposed to SFAs. This approach can facilitate the identification of novel genes and pathways responsive to SFA exposure and regulated by NF-κB. By comparing the transcriptomes of cells with and without NF-κB inhibition, it is possible to ascertain the genes that are directly or indirectly regulated by NF-κB, thus further elucidating the downstream effects of SFA-induced NF-κB activation. Additionally, CRISPR-Cas9 technology can be employed to construct knockout or knockdown models for genes involved in SFA metabolism (such as ELOVL7) or the NF-κB signaling pathway [104–106]. In prostate cancer cells, these models can be utilized to directly assess the functional effects of gene disruption on SFA-induced NF-κB activation and cancer cell proliferation. For instance, after knocking out the ELOVL7 gene in prostate cancer cells and subsequently exposing them to SFAs, the effects on NF-κB activation and tumorigenesis can be observed to determine the necessity of ELOVL7 in SFA-mediated NF-κB activation and tumorigenesis. Moreover, Prostate cancer organoid models can recapitulate the three-dimensional structure and cellular heterogeneity of tumors. They can be used to study the effects of SFAs on NF-κB activation in a more physiological context. Treating organoids with SFAs and observing the activation of NF-κB and subsequent changes in cell behavior (such as proliferation, apoptosis, and differentiation) can help understand the impact of SFAs on the tumor microenvironment and the progression of prostate cancer.

### Translational relevance

Understanding the detailed mechanisms of saturated fatty acid (SFA)-induced NF-κB activation can inform the development of personalized dietary intervention strategies. For instance, if specific SFAs are identified as more potent activators of NF-κB in particular subsets of prostate cancer patients, dietary guidelines can be tailored to limit the intake of foods rich in these SFAs. Furthermore, dietary supplements or functional foods containing bioactive compounds that inhibit SFA-induced NF-κB activation—such as those targeting lipid metabolism or NF-κB signaling—could be developed. Identifying biomarkers associated with SFA-induced NF-κB activation in prostate cancer can facilitate early detection, prognosis prediction, and treatment monitoring. For example, alterations in the levels of specific lipids or NF-κB-regulated genes in blood or urine samples may serve as non-invasive biomarkers. These biomarkers can assist in stratifying patients based on their risk of prostate cancer progression and guide personalized treatment decisions. Insights gained from investigating the SFA-NF-κB-prostate cancer (PCa) relationship can lead to the formulation of novel therapeutic strategies [107]. Targeting lipid metabolism enzymes such as ELOVL7 or components of the NF-κB signaling pathway presents a potential approach. For example, small-molecule inhibitors that block ELOVL7-mediated fatty acid elongation while simultaneously inhibiting NF-κB activation could be developed. Additionally, immunotherapeutic strategies that modulate the inflammatory response associated with SFA-induced NF-κB activation warrant further exploration.

In conclusion, given the critical role of NF - κB in cancer biology and the strong link between SFAs and prostate cancer progression, future research should prioritize the elucidation of the detailed mechanisms by which SFAs activate NF - κB and how this signaling cascade contributes to the development and progression of prostate cancer. Integrating dietary intervention studies with advanced molecular biology techniques will be essential in translating these findings into effective preventive and therapeutic strategies.

## Discussion

The exploration of how saturated fatty acids promote prostate cancer progression via the activation of the NF-κB pathway has provided in - depth insights into the underlying mechanisms of this malignancy. A wealth of epidemiological studies have firmly established a robust association between dietary fat intake, especially from dairy products, and an elevated risk of prostate cancer. For example, Kurahashi et al. (2008) conducted a study that revealed a dose - dependent increase in prostate cancer risk associated with dairy consumption. Specifically, certain saturated fatty acids such as myristic and palmitic acids were found to play a significant role in this process, indicating that these lipids might contribute to cancer progression by influencing key cellular processes.

In recent years, research has delved into the complex role of the NF - κB signaling pathway in prostate cancer biology. Shankar et al. (2017) found that NF - κB is frequently dysregulated in prostate cancer. This dysregulation leads to enhanced tumor cell survival and proliferation. The activation of the NF - κB pathway is closely associated with the inflammatory microenvironment within the tumor, which provides a favorable condition for tumor growth. From a clinical perspective, this suggests that targeting the NF - κB pathway could be a viable therapeutic strategy [108]. For example, developing drugs that inhibit NF - κB activation may help prevent tumor progression and improve patient outcomes.

Notably, the lipogenic gene ELOVL7, which is overexpressed in prostate cancer cells, has emerged as a crucial factor in understanding the metabolic reprogramming related to fatty acid metabolism. Tamura et al. (2009) demonstrated that ELOVL7 is involved in the elongation of saturated very - long - chain fatty acids. This process not only affects the growth of prostate cancer cells but also their survival. In terms of clinical implications, drugs that target ELOVL7 - mediated fatty acid elongation could potentially disrupt the abnormal lipid metabolism in cancer cells, thereby inhibiting tumor growth.

Overall, findings from multiple studies suggest that abnormal lipid metabolism, exacerbated by dietary saturated fats, promotes prostate cancer development through mechanisms involving the NF - κB pathway. This knowledge has significant implications for future prevention and treatment strategies [109]. For prevention, dietary guidelines could be refined to limit the intake of foods high in saturated fatty acids, especially dairy products rich in myristic and palmitic acids. In treatment, therapies that simultaneously target lipid metabolism enzymes like ELOVL7 and the NF - κB signaling pathway could be developed.

Looking ahead, future research should focus on several key areas. First, more in - depth studies are needed to elucidate the precise molecular mechanisms by which saturated fatty acids activate the NF - κB pathway in prostate cancer cells. This could involve identifying the specific receptors and signaling molecules involved in this process. Second, research should explore how different saturated fatty acids interact with the NF - κB pathway and other signaling pathways, such as those related to inflammation and lipid metabolism. Finally, integrating multi - omics data, including lipidomics, transcriptomics, and proteomics, will be essential to comprehensively understand the complex relationship between saturated fatty acids, NF - κB, and prostate cancer. By addressing these research priorities, we can hope to develop more effective preventive and therapeutic approaches for prostate cancer.

## Data Availability

No datasets were generated or analysed during the current study.
